# Impact of Material Stiffness on Medial Arch Support and Stress Redistribution in 3D-Printed Insoles for Adult Acquired Flatfoot Deformity

**DOI:** 10.3390/bioengineering13091010

**Published:** 2026-08-31

**Authors:** Ping-Yen Tsai, Chen-Sheng Chen, Chien-Shun Wang, Ching-Hsuan Chen

**Affiliations:** 1 Department of Physical Therapy, National Yang Ming Chiao Tung University, Taipei 112, Taiwan; baron088880@gmail.com (P.-Y.T.); cschen0623@nycu.edu.tw (C.-S.C.); 2 Department of Orthopedics and Traumatology, Taipei Veterans General Hospital, Taipei 112, Taiwan; 3 Department of Surgery, School of Medicine, National Yang Ming Chiao Tung University, Taipei 112, Taiwan; 4 Department of Mechanical Engineering, Southern Taiwan University of Science and Technology, Tainan City 710, Taiwan; cch1108@gmail.com

**Keywords:** adult-acquired flatfoot deformity, 3D-printed insoles, finite element analysis, material testing, biomechanics

## Abstract

Adult acquired flatfoot deformity (AAFD) results from the collapse of the medial longitudinal arch (MLA), often managed conservatively with foot orthoses. While 3D printing enables rapid and cost-effective production of insoles, the biomechanical impact of different printing materials remains unclear. This study combined material testing, finite element (FE) simulation, and compression testing to evaluate four materials commonly used for 3D-printed insoles: PLA (polylactic acid), resin, EVA (ethylene vinyl acetate), and TPU (thermoplastic polyurethane). Tensile tests provided material properties for FE modeling of balanced standing using a validated healthy foot model. Outcome measures included MLA deformation (navicular drop), plantar stress distribution, and von Mises stress within the insole. A commercial semi-custom insole was tested for comparison. PLA and resin exhibited high stiffness (2228 and 880 MPa) and reduced navicular drop by 55–60%, compared with <30% reductions by EVA and TPU. Plantar stress distribution shifted from forefoot and hindfoot toward the midfoot, with PLA increasing midfoot load share to 38%. Compression testing confirmed that PLA and resin exhibited greater arch support ability than the semi-custom insole within a 10 mm displacement threshold. Material stiffness strongly influenced the biomechanical performance of 3D-printed insoles. Within this static simulation baseline, PLA and resin provided superior arch stabilization and stress redistribution compared with EVA, TPU, and a semi-custom reference. These findings suggest that material choice is critical to optimizing 3D-printed orthotic support, providing a valuable biomechanical baseline to guide future orthotic designs for flatfoot.

## 1. Introduction

Adult acquired flatfoot deformity (AAFD) arises from tendon and ligament insufficiencies that collapse the medial longitudinal arch (MLA) and impair function [1]. Posterior tibialis tendon dysfunction (PTTD) is a well-recognized cause, but damage to other soft tissues, such as the spring ligament, deltoid ligament, and plantar fascia, is also common [2]. AAFD is prevalent in older adults and often compromises gait and daily activities, making conservative treatment the first choice before surgery [3,4,5].

Foot orthoses are widely used in conservative care. They can reduce excessive pronation, relieve pain, and improve function [6,7,8,9]. Among them, 3D printing has gained attention for orthosis development because it is fast, repeatable, and cost-effective [10,11]. Studies have shown that 3D-printed insoles can relieve pain and redistribute plantar loads, with both short- and long-term benefits [12,13,14,15,16]. Although 3D-printed insoles offer superior anatomical conformity and localized stiffness control, their biomechanical outcomes remain highly inconsistent [7,17]. Prior studies report conflicting results regarding dynamic plantar pressure reduction and center of pressure trajectory shifts during walking [12,14]. Differences in diagnosis, insole design, printing methods, and especially material selection may explain this variability.

Finite element (FE) modeling has become a key tool to investigate in-foot biomechanics. Existing FE studies have examined how arch height, stance-phase loading, and muscle or tendon conditions affect joint forces [18,19,20]. More advanced models have added ligament and muscle details to explore AAFD-related changes [21]. A few studies have also applied FE models to orthoses, focusing on posting angles or arch height in 3D-printed insoles [22]. Yet, no study has systematically compared the biomechanical impact of different 3D-printing materials, leaving little guidance for material-specific orthotic design. Additionally, the traditional commercial insole should be compared to the 3D-printed insole in terms of stiffness to determine its performance in preventing arch collapse.

This study fills that gap by combining 3D printing material testing, FE analysis, and insole compression testing. This study evaluated how material choice in 3D-printed insoles for AAFD influences (1) MLA deformation (navicular drop) and (2) plantar stress distribution. Linking experimental properties to the FE model predictions provides evidence to guide material selection for customized 3D-printed orthoses.

## 2. Materials and Methods

### 2.1. 3D Printing Material Test

Four commonly used 3D-printing materials for foot orthoses were investigated: PLA (polylactic acid), resin, EVA (ethylene vinyl acetate), and TPU (thermoplastic polyurethane) [23,24,25,26,27,28]. For each material, five ASTM D638-14 Type I specimens were printed. PLA (Shore ~82D) and TPU (Shore 90A) specimens were printed using a Bambu X1 Carbon printer (Bambu Lab Co., Ltd., Shenzhen, China), while EVA specimens (Shore ~90A) were fabricated using a Creality CR10V3 printer (Creality 3D Technology Co., Ltd., Shenzhen, China). These fused deposition modeling (FDM) specimens were printed at a 0.2 mm layer height with 100% concentric infill density, aligning tool-paths parallel to the tensile axis to minimize anisotropy. The nozzle temperatures ranged from 210 to 230 °C, and the extrusion widths were set between 0.4 and 0.5 mm. Other printing parameters, including platform temperature and print speed, are summarized in the Supplementary Materials. Resin specimens (Shore 77D) were printed using an LCD-based Phrozen Sonic Mighty 8K printer (Phrozen Tech Co., Ltd., Hsinchu, Taiwan) at a 0.05 mm layer height and post-cured using a UV lamp (48 W) for 15 min per side.

Uniaxial tensile tests were performed using a universal testing machine (HT-2402, Hung Ta Instrument Co., Ltd., Taichung, Taiwan) equipped with a two-point extensometer (HT-9106A, Hung Ta Instrument Co., Ltd., Taichung, Taiwan; gauge length: 50 mm). The crosshead speed was 5 mm/min, and tests were terminated either at specimen fracture or at 40% strain to protect the extensometer. For the rigid plastics (PLA and resin), Young’s modulus was determined as the chord modulus within the 0.05–0.25% strain interval, and yield stress was calculated using the 0.2% offset method. For the compliant elastomers (EVA and TPU) lacking a distinct linear-elastic region, stiffness was determined as the tangent modulus at 5% strain. This prevents potential overestimation by the chord modulus and accurately captures their mechanical behavior under functional in-shoe loading conditions. The engineering yield stress was defined as the stress corresponding to 5% strain, as the ASTM D412. Force–displacement data from the tensile tests were converted to stress–strain curves, and the mean ± SD of the calculated parameters were used as input for the FE models (Table 1) [25,29,30,31].

### 2.2. FE Modeling

#### 2.2.1. Foot and Ground Model

**Figure 1:**
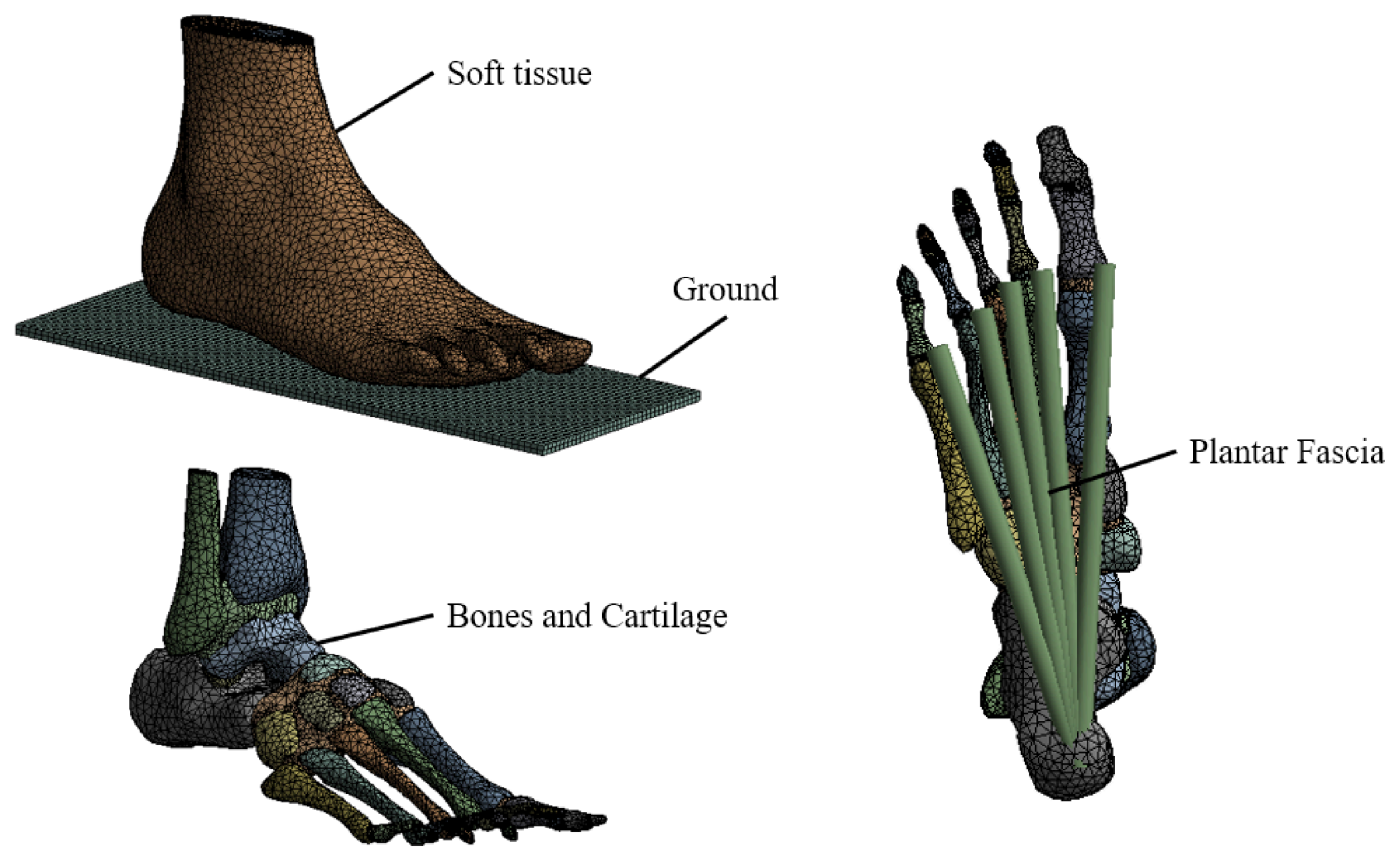
Barefoot finite element model by Chen et al. [28] with boundary and loading conditions.

The foot FE model used in this study was directly adopted from the validated model developed by Chen et al. [28], which represents a healthy adult male (177 cm, 70 kg) in a neutral unloaded position (Figure 1). The model comprises 28 bones, articular cartilage, encapsulating soft tissue, and the plantar fascia, all of which are represented by five truss elements (Table 2). A rigid plate was defined as the ground surface. The foot FE model was reconstructed using ANSYS 2021 (ANSYS, Inc., Canonsburg, PA, USA). This FE model has been previously validated against plantar pressure measurements obtained with the Pedar-X system (novel GmbH, Munich, Germany), showing good agreement in average and peak pressures across the forefoot, midfoot, and hindfoot regions [27]. Therefore, it was adopted as the baseline barefoot condition for subsequent insole analyses in the present study.

#### 2.2.2. Insole Models

**Figure 2:**
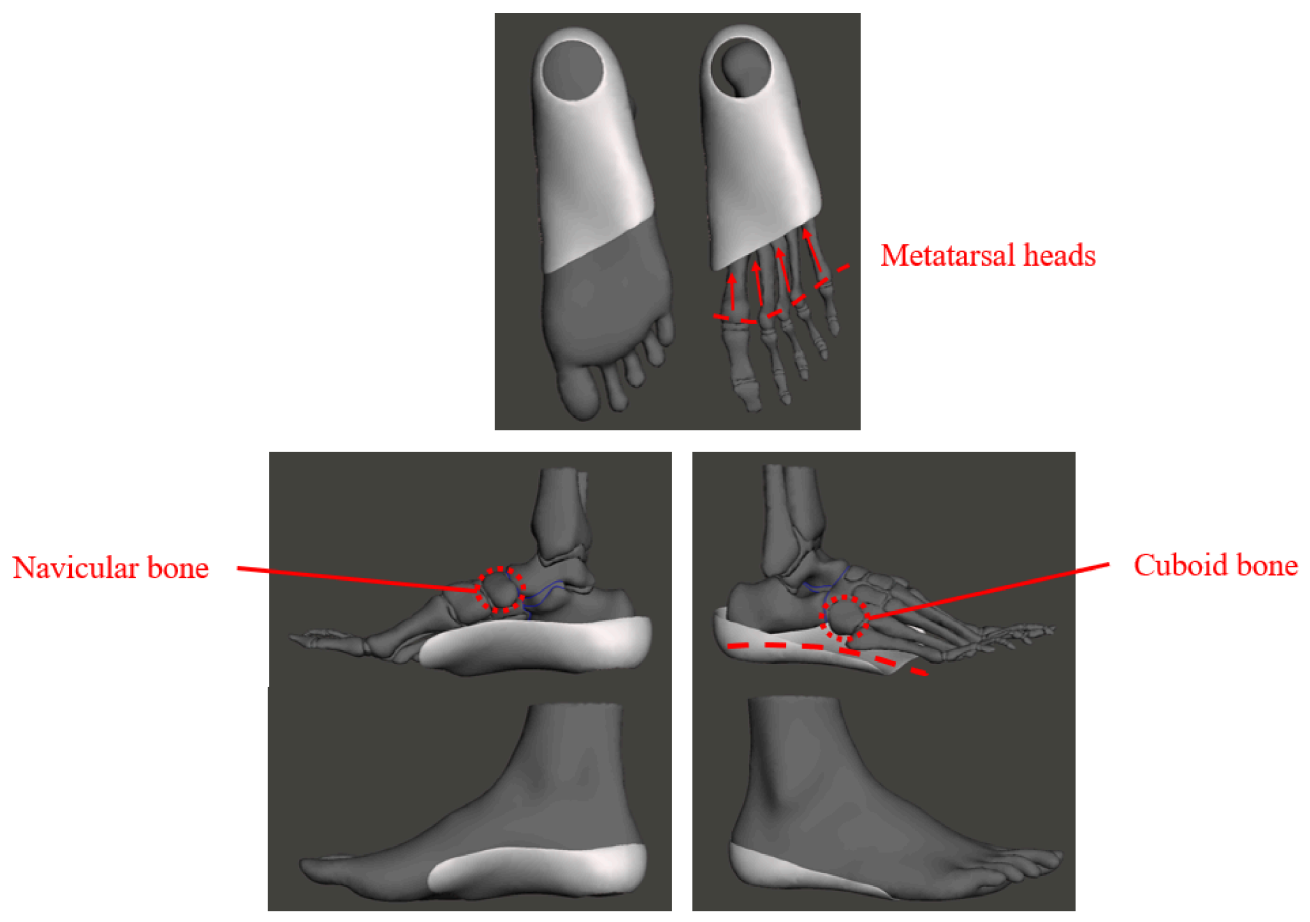
Construction steps of insole geometry using Meshmixer [19]. Note: The red line indicates the regions where the insole supports the medial and lateral longitudinal arches, positioned slightly beneath the navicular and cuboid bones while terminating proximal to the metatarsal heads to maintain the windlass mechanism.

Custom insole geometry was created by extracting the plantar surface (¾ length) from the barefoot model using Meshmixer (Version 3.5.474, Autodesk, Inc., San Rafael, CA, USA) and extruding it to a 2 mm thickness. Key modifications included a heel recess for the fat pad and local smoothing to remove sharp edges (Figure 2). To preserve the normal windlass mechanism during gait, the insole was designed to end just proximal to the metatarsal heads. It aimed to provide appropriate support to both the medial and lateral longitudinal arches, positioned slightly below the navicular and cuboid bones. Four insole models were generated, each assigned with an experimentally derived Young’s modulus in our mechanical test, but the Poisson’s ratios were adopted from the literature [25,29,30,31] (Table 1). The complete FE foot model consisted of 51,058 nodes and 154,217 elements. A total of five FE models were constructed: barefoot, PLA insole, resin insole, EVA insole, and TPU insole.

#### 2.2.3. Boundary and Loading Conditions

**Figure 3:**
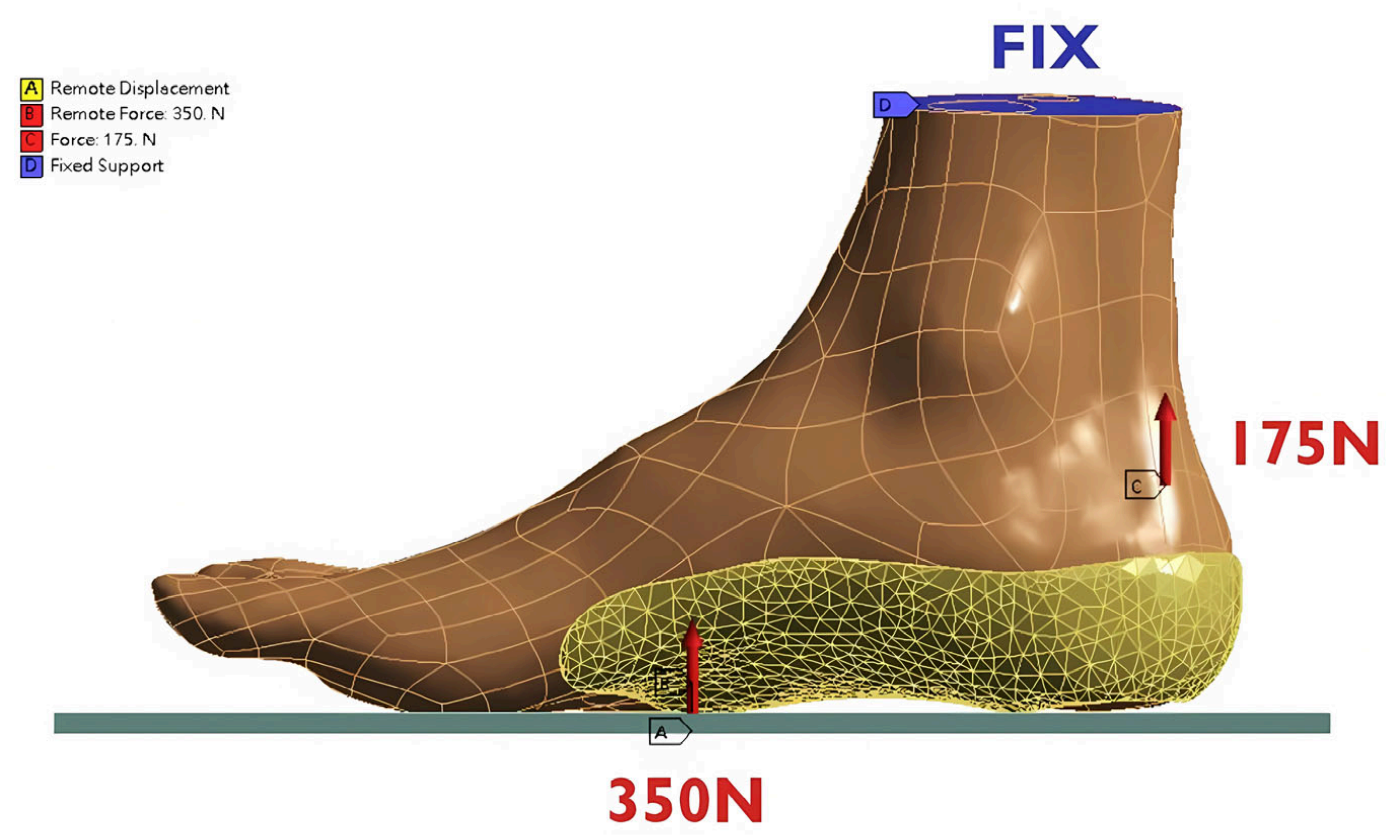
Boundary and loading conditions of the finite element model.

Balanced standing was simulated, so the superior surfaces of the tibia, fibula, and soft tissue were fixed. A ground reaction force of 350 N (representing half of the body weight for a 70 kg subject) was applied vertically to the rigid ground plate, and a 175 N Achilles tendon force was applied to the calcaneus, following Chen et al. [28] (Figure 3). Contact conditions were defined: bonded interactions were assigned to all internal foot structures and to the foot–insole interface, whereas frictionless contacts were specified for the foot–ground and insole–ground interfaces.

#### 2.2.4. Biomechanical Analysis

The biomechanical outcomes included medial arch deformation, plantar stress distribution, and stress within the insole and bones. MLA deformation was expressed as a navicular drop from the relative vertical displacement between the navicular medial arch and the ground. Plantar stress distribution was obtained as principal stress of the soft tissue, with average values calculated for the forefoot, midfoot, and hindfoot after subdividing the plantar surface according to the Lisfranc and Chopart joints. In addition, von Mises stress of the foot bones was evaluated.

### 2.3. Compression Testing of the Medial Arch in the Insoles

**Figure 4:**
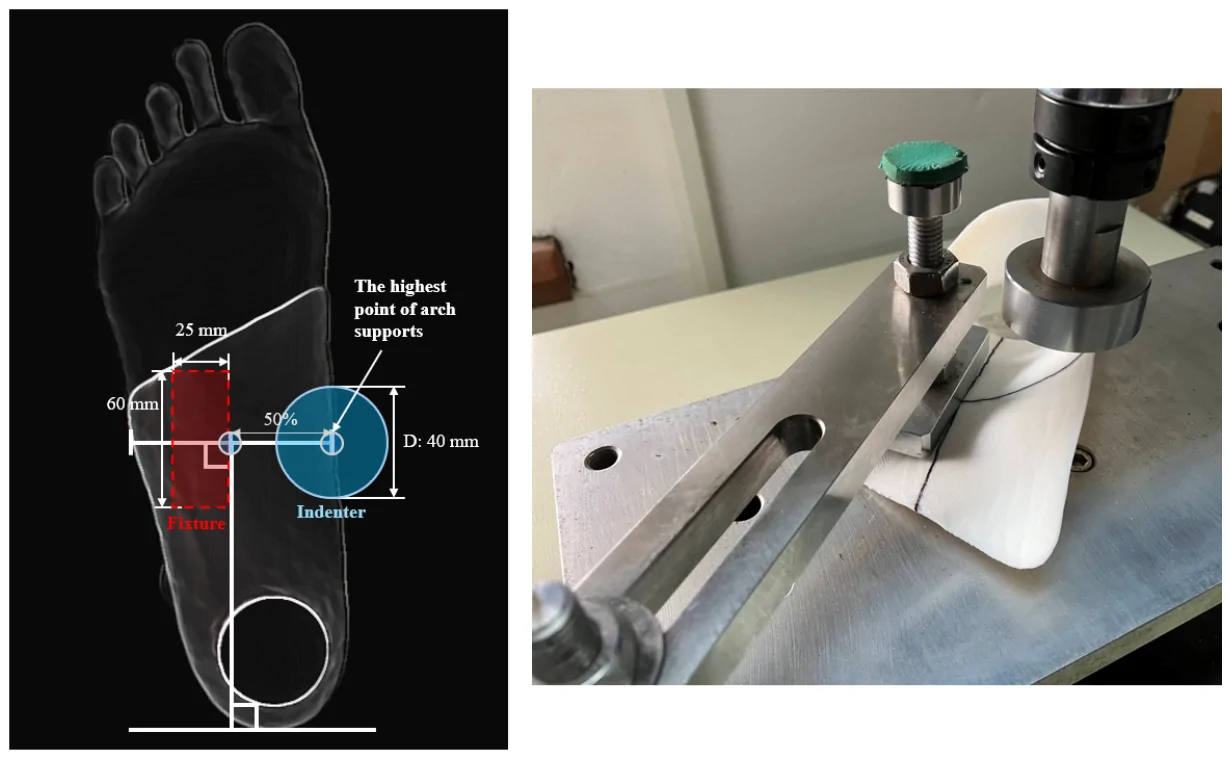
Compression test of arch support in 3D printed insoles [12].

To ascertain the difference between the 3D printed insole and the current commercial insole, a prefabricated semi-custom insole (MBF G2, Meibin Orthotics Co., Ltd., Taichung, Taiwan) was included for comparison of arch support. The medial arch height of the commercial semi-custom insole was adjusted through heat molding to match the 3D printed insoles (30.5 mm). Compression tests were conducted in accordance with the previous study [12]. Each insole (*n* = 6) was positioned in a testing machine with the heel stabilized by a rectangular fixture (60 × 25 mm). A cylindrical indenter (diameter 40 mm) applied load vertically at the highest point of the medial arch at a rate of 105 N/min by the testing machine (HT-2402, Hung Ta Instrument Co., Ltd., Taichung, Taiwan) (Figure 4). Force–displacement data were recorded, and stiffness was calculated as the secant stiffness at an arch displacement of 10mm. In the navicular drop test, an abnormal arch collapse is defined as the arch of the foot changing by more than 10 mm [32].

## 3. Results

### 3.1. FE Analysis

#### 3.1.1. Medial Longitudinal Arch Deformation

**Figure 5:**
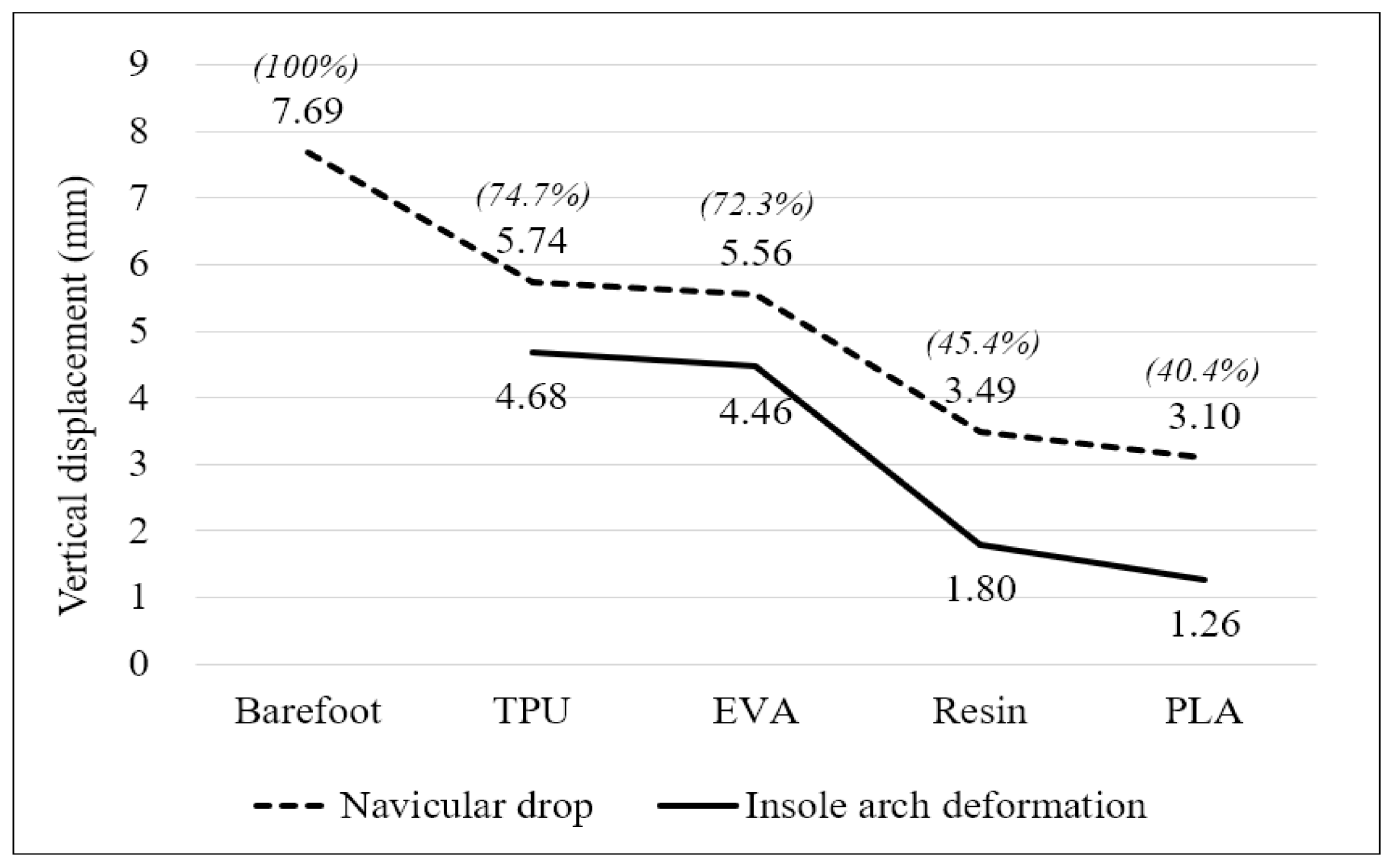
Medial longitudinal arch deformation in FE analysis.

Navicular drop was greatest in the barefoot condition (7.69 mm). Insole models exhibited reduced deformation with increasing material stiffness, with PLA and resin achieving the largest reductions (59.6% and 54.6%), while EVA and TPU provided more modest reductions (27.7% and 25.3%), respectively (Figure 5).

#### 3.1.2. Foot and Insole Stress

**Figure 6:**
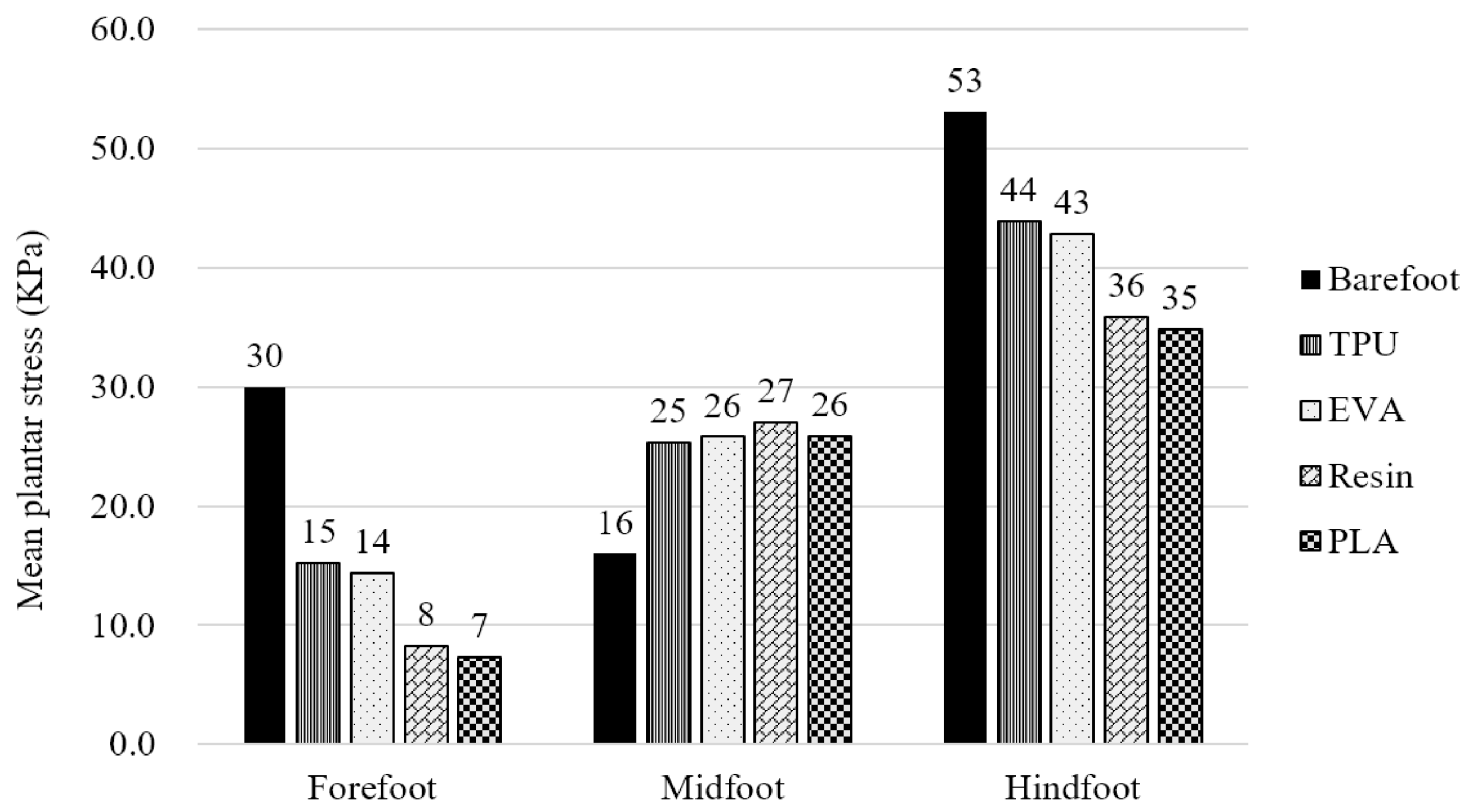
Average plantar stress in forefoot, midfoot, and hindfoot.

**Figure 7:**
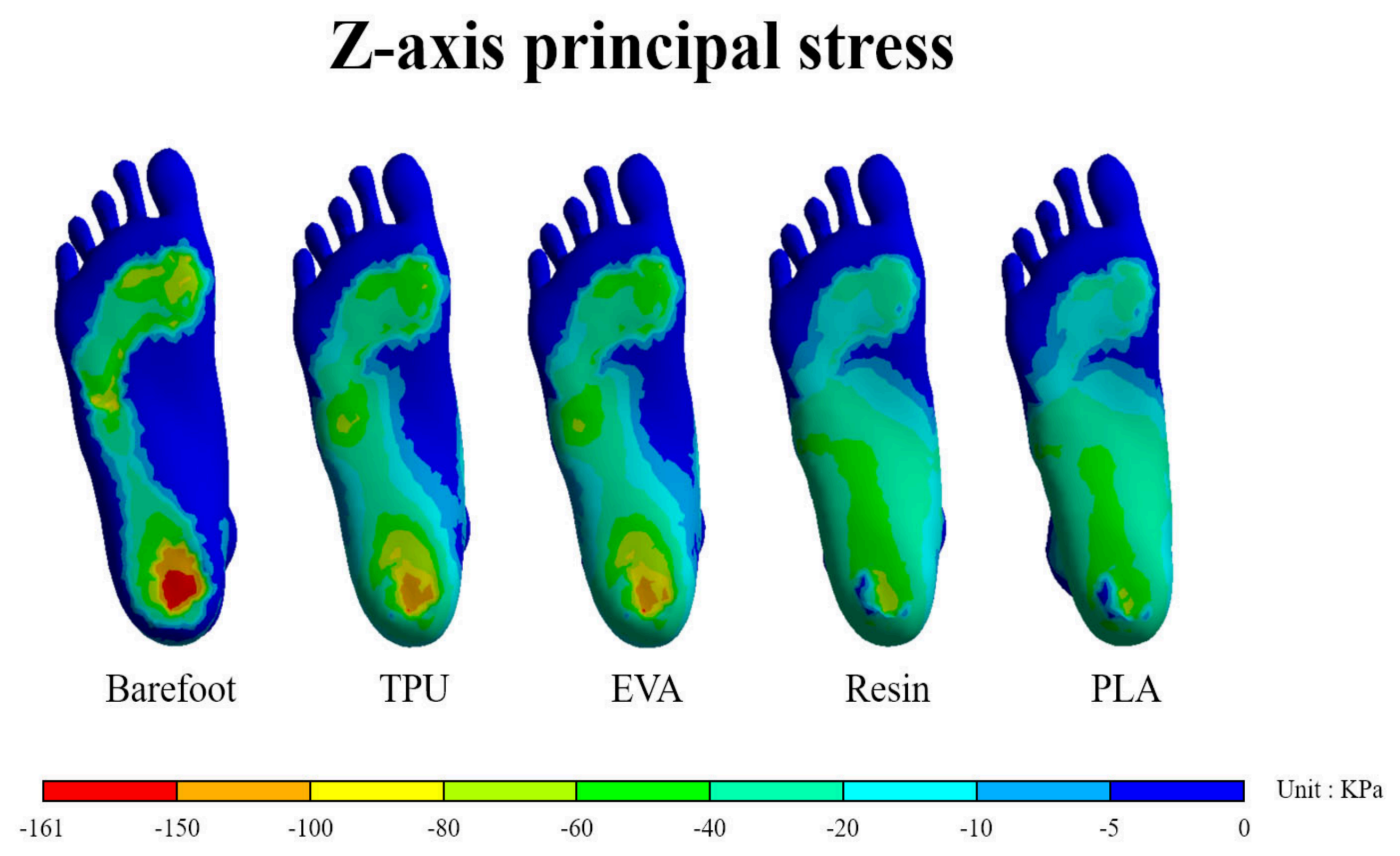
Plantar stress distribution (principal stress in z-axis).

**Figure 8:**
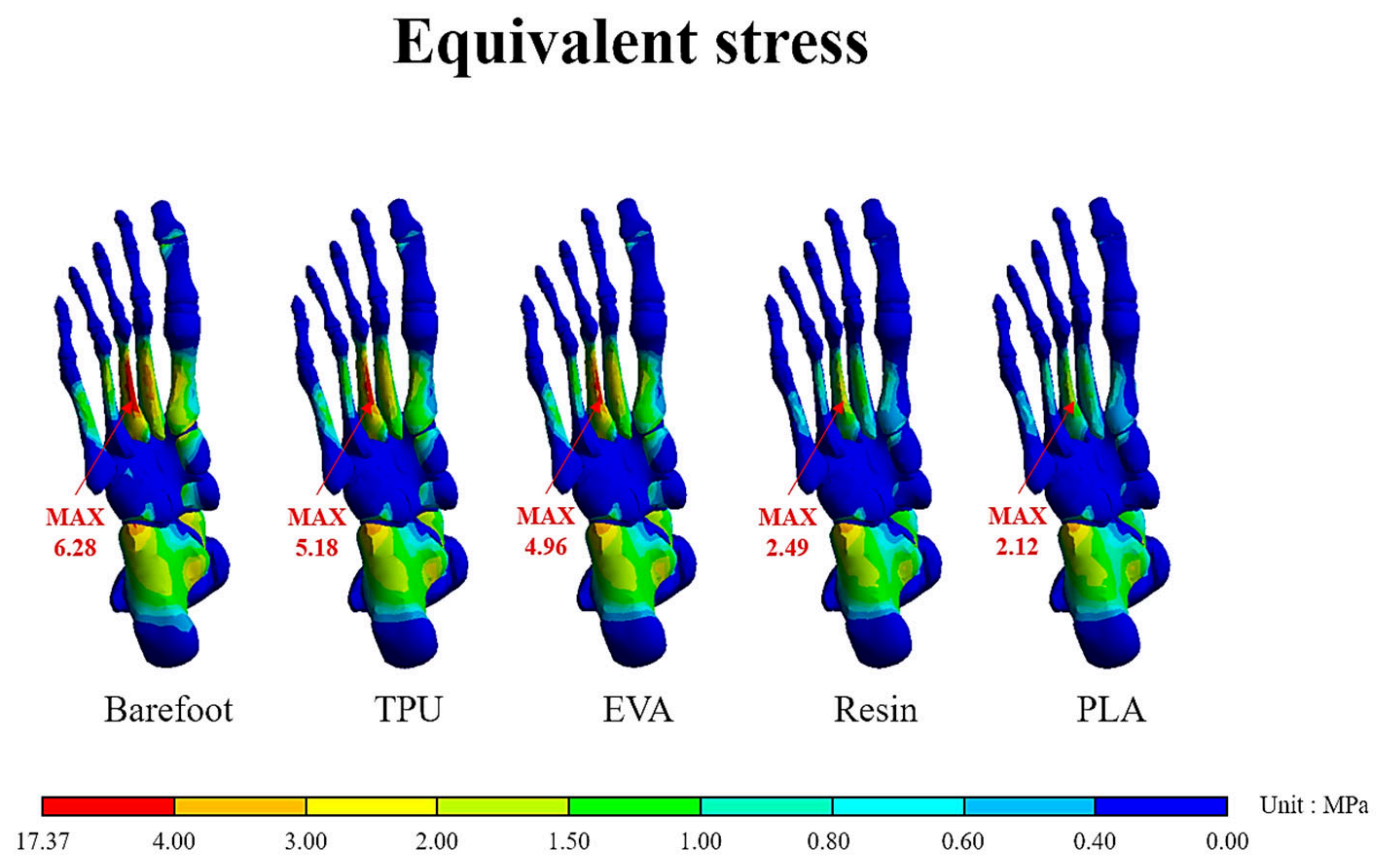
Equivalent stress in the foot bones and a peak at the third metatarsal.

Barefoot loading resulted in higher stresses in the forefoot and hindfoot than in the midfoot. Insole models reversed this pattern, shifting load toward the midfoot (Figure 6). Stiffer insoles produced greater redistribution: PLA reduced forefoot stress by 77% compared to barefoot, while TPU reduced it by 50%. Midfoot stress share increased from 20.4% (barefoot) to 30.0% with EVA and 38.0% with PLA. In addition, stiffer materials increased the midfoot load-bearing area and further dispersed hindfoot stress, with PLA and resin showing markedly lower hindfoot stresses than the barefoot condition (Figure 7). Consistent with these results, equivalent stress in the bones was highest in the third metatarsal under barefoot conditions (6.28 MPa) but decreased when insoles were worn, especially with PLA and resin (Figure 8).

**Figure 9:**
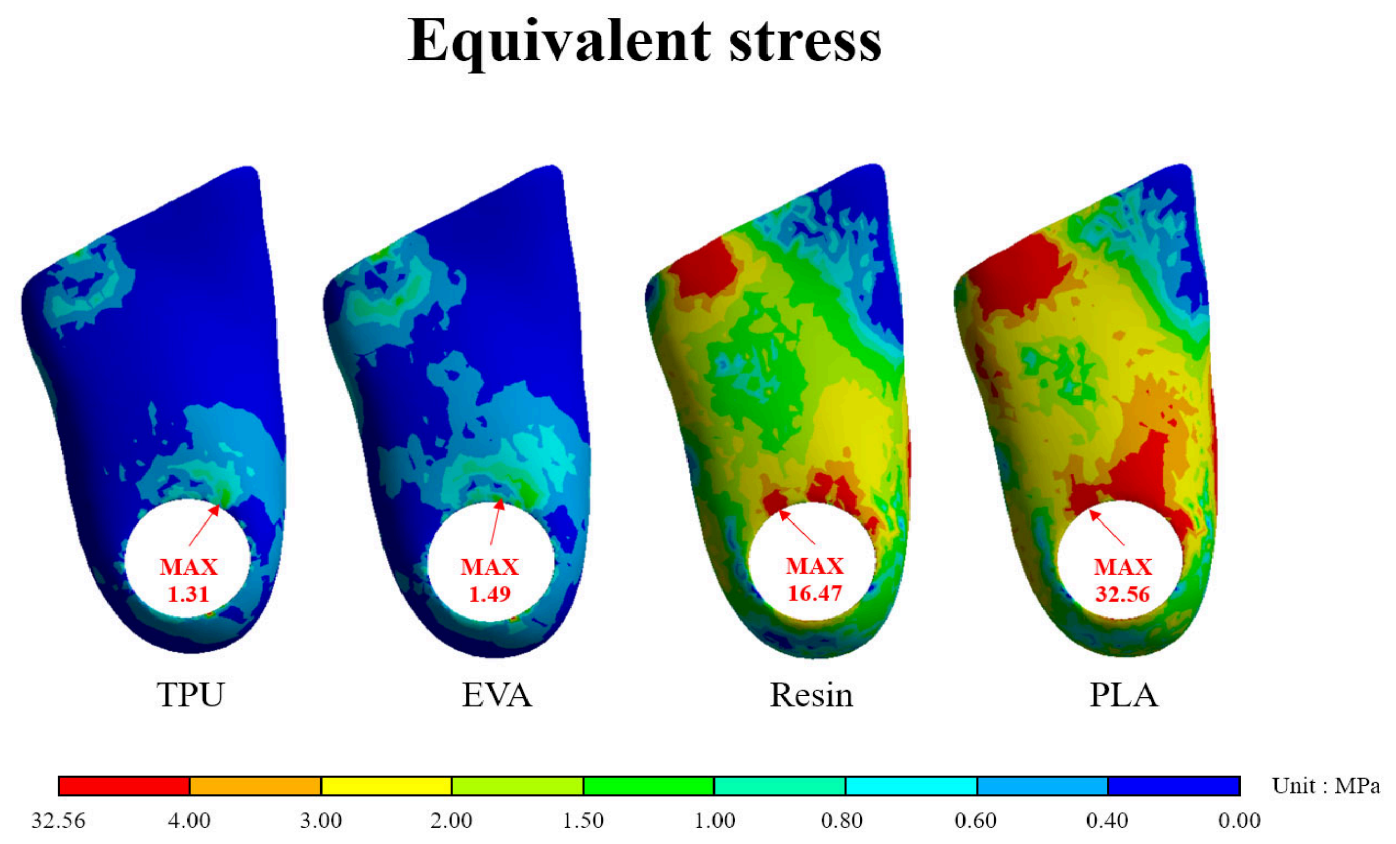
Equivalent stress distribution within insoles.

Von Mises stress within the insoles varied widely by material stiffness. TPU and EVA exhibited low peak stresses (1.31 and 1.49 MPa), whereas resin and PLA reached substantially higher values (16.47 and 32.56 MPa), concentrated near the anterior edge of the heel cutout (Figure 9).

### 3.2. Real Insole Compression Testing

**Figure 10:**
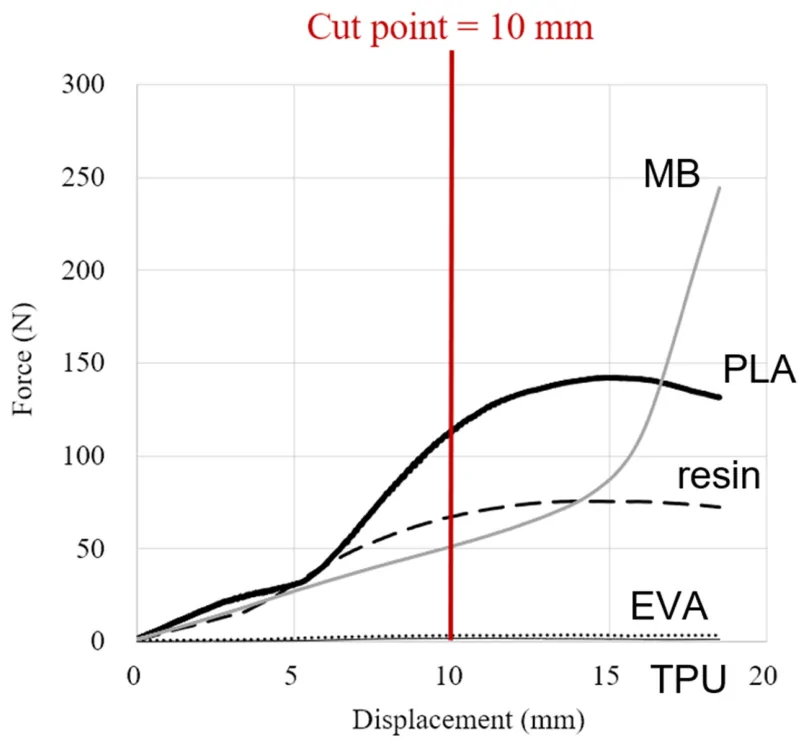
Compression test of the medial arch among the five insoles.

To assess the difference between the 3DP insole and commercial insole, the stiffness of arch support was tested. All real insoles tolerated compression without material failure. Compared at 10 mm displacement, resin (6.8 N/mm) and PLA (11.1 N/mm) provided superior stiffness compared to the commercial semi-custom insole MB (5.15 N/mm), while EVA (0.36 N/mm) and TPU (0.18 N/mm) offered minimal support (3–7%) (Figure 10).

## 4. Discussion

FE simulation confirmed that stiffer insoles substantially reduced navicular drop, consistent with previous randomized controlled trials and FE studies [33,34,35,36,37]. PLA and resin reduced navicular displacement by 4–5 mm, whereas EVA and TPU achieved less than 2 mm reduction, which may not reach clinically meaningful thresholds [35,36,37]. Unlike most clinical trials that assessed barefoot conditions, this study evaluated navicular displacement while the insoles were worn, directly reflecting their mechanical support [31,32,33,34,35,36,37], thereby reflecting the direct mechanical support of the insole. This approach demonstrates that FE-based simulation of navicular drop test not only provides a more precise understanding of arch stabilization but also highlights material stiffness as a key determinant of arch support [38,39,40].

Plantar stress distribution further supported these findings. Barefoot stance produced dominant loading in the forefoot and hindfoot, with minimal midfoot contribution, whereas all insole models shifted stresses toward the midfoot. PLA reduced forefoot and hindfoot stresses by 77% and 34%, respectively, while TPU achieved 50% and 17%. The midfoot share increased from 20.4% (barefoot) to 30.0% with EVA and 38.0% with PLA. Stiffer insoles also expanded the midfoot contact area and dispersed hindfoot loading, particularly around the heel cutout, a design feature relevant to reducing fat pad overload [41]. Equivalent stress analysis of the bones further indicated that barefoot loading concentrated stress at the third metatarsal head, whereas PLA and resin insoles reduced this peak, consistent with the reduction in forefoot plantar stresses [42]. Clinically, such redistribution may prevent first-ray hypermobility [43] and attenuate risks of heel pain syndromes. These results align with in-shoe pressure studies [44] and FE simulations [42], though some reports observed increased forefoot loading with stiffer insoles [45], likely due to softer material assumptions or conventional geometries. Overall, stiffer 3D-printed insoles provided superior redistribution of plantar stresses, reinforcing their clinical potential for flatfoot management. Although this study specifically targeted insoles designed for AAFD, the observed biomechanical mechanisms—arch stabilization and stress redistribution—may also help explain potential benefits in related conditions such as fat pad syndrome and first-ray hypermobility.

The insole-level analyses provided further insights into material performance and durability. Von Mises stress in PLA and resin insoles exceeded their measured yield strengths, indicating a risk of plastic deformation and potential material failure under repetitive loading conditions. This pattern is consistent with clinical observations that harder orthoses, while effective for arch support, may fracture after prolonged use. Stress was concentrated at the heel aperture, a region previously reported as a common failure site [37], though such apertures remain clinically beneficial for dispersing plantar pressure and reducing heel impact. In parallel, compression testing revealed that PLA and resin insoles provided greater stiffness than the commonly used prefabricated semi-custom MB insole within the clinically relevant navicular drop range (≤10 mm). This comparison evaluates the overall performance of the orthotic designs rather than isolating raw material superiority as the groups differ in both geometry and material construction. Specifically, while foam-based prefabricated insoles are clinically valued for cushioning, rigid 3D-printed designs with customized thin-shell geometries can surpass traditional prefabricated models in providing MLA support under moderate deformation conditions. In contrast, EVA and TPU insoles offered negligible arch support in compression testing, reflecting their low stiffness and thin-shell geometry. Together, these findings highlight a trade-off: while stiffer printed insoles (PLA and resin) provide superior arch stabilization within physiological ranges, they also accumulate higher internal stresses and are more prone to material fatigue or fracture, a limitation that future design optimization must address.

Alongside finite element (FE) modeling, other computational and design tools are widely used to evaluate foot biomechanics. Multi-body dynamics (MBD) simulations effectively predict joint kinematics, segment accelerations, and muscle forces over complete gait cycles [46,47]. Additionally, lattice and generative design tools automate structural modifications to enable localized stiffness tuning and lightweighting [17,48]. However, MBD simplifies anatomical structures as rigid bodies, failing to capture internal stresses and strains within soft tissues. Similarly, design optimizers focus primarily on macroscopic material layouts. FE modeling remains uniquely advantageous because it reconstructs subject-specific 3D geometries of bones, cartilage, and soft tissues. This anatomical detail enables the evaluation of internal load sharing and localized tissue strains. These insights are essential for assessing the biomechanical efficacy of orthotic interventions.

This study has several methodological considerations. First, the FE model was reconstructed from a single healthy adult foot, which allowed controlled material comparisons but does not capture inter-subject variability in arch height, body weight, or sex, nor the pathological features of AAFD. Therefore, the outcomes serve as a biomechanical baseline, requiring caution when applying them to symptomatic AAFD patients. Second, all insole materials were modeled as linear isotropic materials for computational stability. Under static standing conditions, the overall insole deformation is relatively small and localized, and this simplified approach aligns with previous foot orthosis FE investigations [22]. Additionally, while peak stresses at contact boundaries occasionally exceeded the polymer yield strengths, these localized extremes represent numerical concentrations rather than structural failure. Third, the foot–insole interface was assumed to be bonded, and ground contact was modeled as frictionless to facilitate model convergence. While these simplifications are common in static FE models, they neglect boundary shear sliding and tangential resistance. This may lead to an underestimation of local shear stresses and bias the predicted stress redistribution. Fourth, although mechanical experiments reported mean and standard deviation values, formal inferential statistics were not performed as these datasets primarily served as inputs for our simulations. Finally, this static analysis did not evaluate cyclic or fatigue loading; thus, long-term wearability and actual material fracture with repetitive use require further validation.

Despite these simplifications, the consistent ranking of materials across FE simulation and compression testing supports the robustness of the finding that stiffer materials (PLA and resin) provide superior arch stabilization compared with softer insoles. This highlights material stiffness as a dominant factor in orthotic design, with potential clinical implications for conservative management of AAFD. To obtain comprehensive clinical data, human-subject experiments are planned in our next research phase to evaluate dynamic gait patterns, in-shoe plantar pressure, and actual navicular drop. Furthermore, future computational studies should incorporate subject-specific flatfoot geometries and fatigue testing to better capture clinical scenarios and long-term durability.

## 5. Conclusions

This study systematically compared four 3D-printing materials for insoles by combining material testing, FE modeling, and compression testing. PLA and resin, characterized by high stiffness, provided the greatest medial arch stabilization, reducing navicular drop by 55% while also shifting plantar stresses toward the midfoot. TPU and EVA, by contrast, offered minimal structural support and negligible compression stiffness. Given the need to prevent medial arch collapse, this static biomechanical evaluation suggests that PLA and resin are promising candidates for customized 3D-printed insoles. These findings provide a valuable biomechanical baseline to guide future designs for conservative flatfoot management.

## Figures and Tables

**Table 1:** Mechanical properties of 3D-printed insoles.

	Young’s Modulus (MPa)	Yield Stress (MPa)	Poisson Ratio
PLA	2227.76 ± 39.45	29.96 ± 0.31	0.33 [28]
Resin	879.79 ± 47.35	12.12 ± 0.02	0.41 [29]
EVA	24.69 ± 7.52	1.92 ± 0.04	0.45 [30]
TPU	20.24 ± 3.19	1.39 ± 0.03	0.49 [24]

**Table 2:** Material properties and element types of the barefoot model by Chen et al. [28].

	Elastic Modulus (MPa)	Poisson Ratio	Element Types	Cross-Sectional Area (mm^2^)
Bone	7300	0.3	Solid 185	-
Cartilage	1	0.4	Solid 185	-
Plantar fascia	350	-	Link 180, tension-only	290.7/5 = 58.14
Soft tissue and skin	Hyperelastic (polynomial form) C_10_ = 0.08556, C_01_ = −0.05841, C_20_ = −0.039, C_11_ = −0.02319, C_02_ = 0.00851 (MPa); D_1_ = 3.65273, D_2_ = 0 (MPa^−1^)		Solid 285	-

## Data Availability

The original contributions presented in the study are included in the article/Supplementary Materials; further inquiries can be directed to the corresponding author.

## Supplementary Materials

The following supporting information can be downloaded at: https://www.mdpi.com/article/10.3390/bioengineering13091010/s1, Table S1: 3D Printing Parameters of PLA, TPU, and EVA; Table S2. Resin Printing Parameters.
