# Supplementary material for: Impact of Material Stiffness on Medial Arch Support and Stress Redistribution in 3D-Printed Insoles for Adult Acquired Flatfoot Deformity
Source: Bioengineering (Basel). 2026 Aug 31;13(9):1010. doi: 10.3390/bioengineering13091010 (PMC13603734; doi:10.3390/bioengineering13091010)
Supplement: Supplementary file 1 [file bioengineering-13-01010-s001.zip › bioengineering-4486336-supplementary.pdf]

Supplementary Materials

Table S1. 3D Printing Parameters of PLA, TPU, and EVA

	PLA	TPU	EVA
Nozzle temperature (°C)	220	230	210
Platform temperature (°C)	35	30	65
Layer height (mm)	0.2	0.2	0.2
Extrusion width (mm)	0.42~0.5	0.42~0.5	0.4
Number of outer walls (layers)	2	2	2
Number of top layers (layers)	2	2	2
Number of bottom layers (layers)	2	2	2
Infill density	100%	100%	100%
Travel speed (mm/s)	500	500	150
Printing speed – first layer (infill) (mm/s)	105	105	20
Printing speed – first layer (outer wall) (mm/s)	50	50	20
Printing speed – other outer walls (mm/s)	200	200	20
Printing speed – other inner walls (mm/s)	300	300	20
Printing speed – infill (mm/s)	250~270	250~270	40
Printing speed – top layers (mm/s)	200	200	20~40

Table S2. Resin Printing Parameters

Layer height (mm)	0.05	Bottom lift distance (mm)	8
Number of bottom layers (layers)	6	Lift distance (mm)	8
Exposure time (s)	9	Bottom retract distance (mm)	8
Bottom exposure time (s)	40	Retract distance (mm)	8
Number of transition layers (layers)	6	Bottom lift speed (mm/min)	60
Transition layer delay (s)	4.43	Lift speed (mm/min)	60
Pre-lift resting time (s)	0/0	Bottom retract speed (mm/min)	150
Post-retraction resting time (s)	4	Retract speed (mm/min)	150
